# Nomogram to predict the incidence of new-onset heart failure after acute coronary syndrome among women

**DOI:** 10.3389/fcvm.2023.1131813

**Published:** 2023-03-24

**Authors:** Qiqi Yan, Lifang Ye, Qinggang Zhang, Jikai Song, Xin Zhang, Liuyang Wu, Lihong Wang

**Affiliations:** ^1^ Heart Center, Department of Cardiovascular Medicine, Zhejiang Provincial People’s Hospital (Affiliated People’s Hospital, Hangzhou Medical College), Hangzhou, China; ^2^ The Second School of Clinical Medicine, Zhejiang Chinese Medical University, Hangzhou, China; ^3^ Zhejiang Provincial People's Hospital, Qingdao University, Hangzhou, China

**Keywords:** acute coronary syndrome, heart failure, nomogram, women, 10-fold cross-validation

## Abstract

**Background:**

Although great progress has been made in caring for patients with acute coronary syndrome (ACS), the incidence of heart failure (HF) after discharge remains high after ACS.

**Aims:**

We aimed to investigate the risk predictors for new-onset HF and build a simple nomogram to optimize the clinical management of female patients.

**Methods:**

The clinical data of 319 female patients with ACS between January 1, 2021 and January 1, 2022, were obtained from the Zhejiang Provincial People’s Hospital. Multivariate logistic regression analysis was carried out to build the prediction model among all participants and then verified by 10-fold cross-validation. The discrimination, calibration, and clinical usefulness of the prediction model were assessed using receiver operating characteristic curve, calibration curve, and decision curve analyses.

**Results:**

This study analyzed 15 potential independent risk predictors of new-onset HF in 319 female patients with ACS. The incidence of HF onset was 23.2%. The following 5 independent risk predictors were filtered out as most relevant for predicting 12-month HF onset: left ventricular ejection fraction ≤ 60.5%, high-density lipoprotein ≤ 1.055 mmol/L, human epididymal protein 4 > 69.6 pmol/L, creatinine > 71.95 µmol/L, and diagnosis of myocardial infarction (MI).

**Conclusion:**

Our nomogram, which used five easily obtained clinical variables, could be a useful tool to help identify female individuals with ACS who are at high risk of developing HF after discharge and facilitate communication between female patients and physicians.

## Introduction

Although great progress has been made in the care of patients with acute coronary syndrome (ACS), the incidence of heart failure (HF) at discharge remains high after ACS (1). In addition, sex differences in new-onset HF after ACS are noteworthy. A recent prospective study reported that whether under unadjusted analysis or multivariate adjustment, women carried a higher risk of HF onset than men (2, 3). Moreover, compared to men, women with coronary artery disease will be older and have higher risk of hypertension, diabetes, and congestive HF (4). However, as we know, the symptoms and signs of HF provide limited diagnostic accuracy (5), while female patients present a more atypical clinical HF status (6, 7). Additionally, it has been reported that nearly two out of three female patients with coronary artery disease show persistent symptoms and ischemic signals, but coronary angiography (CAG) shows no obstructive coronary lesions, which significantly affects their prognosis (8, 9). Therefore, we aimed to detect the risk predictors for new-onset HF and build a simple nomogram to optimize the clinical management of female patients.

## Materials and methods

### Study patients

We conducted a single-center, retrospective, observational cohort study in Zhejiang Provincial People's Hospital (Hang Zhou, China). A total of 319 female patients who experienced ACS and underwent CAG from January 1, 2021 to January 1, 2022, were included in this study and followed up for one year by telephone, and all subsequent hospitalizations, emergency admissions, and outpatient visits were reviewed. The development of a new-onset HF event (New York Heart Association heart failure classes ranging from II to IV) was our main observation outcome. When screening the included population, we excluded patients with heart failure or those who had previously underwent percutaneous coronary intervention (PCI) or coronary artery bypass grafting. In addition, patients were diagnosed with ACS, including myocardial infarction (MI) and unstable angina (UA), according to the criteria of the 2020 ESC Guidelines (10), while HF was diagnosed according to the criteria of the 2021 ESC Guidelines (11). This study was conducted in accordance with the principles of the Declaration of Helsinki. The study protocol was reviewed and approved by the Institutional Review Board of Zhejiang Provincial People's Hospital (Hangzhou, China), registration number: QT2022337. Informed consent was waived due to the retrospective nature of the study.

### Data collection and variables

Clinical information was obtained from the patients' medical records, including demographics [age, body mass index (BMI), history of smoking, and alcohol consumption]; history of hypertension and type 2 diabetes; vital signs at admission (pulse, systolic blood pressure (SBP), and diastolic blood pressure (DBP)); left ventricular ejection fraction (LVEF); administration of angiotensin-converting enzyme inhibitor or angiotensin receptor blocker (ACEI or ARB), calcium channel blocker (CCB), oral antidiabetics, and insulin 3 months before admission; results of coronary angiography; whether PCI was performed; and medications at discharge [aspirin, clopidogrel, ticagrelor, ACEI or ARB, beta-blocker, statins, thiazide diuretic, and sodium-glucose cotransporter-2 inhibitor (SGLT2i)]. In addition, we collected the results of patients' laboratory findings during hospitalization, including hemoglobin, total cholesterol (TC), triglyceride (TG), high-density lipoprotein cholesterol (HDL-C), low-density lipoprotein cholesterol (LDL-C), glycosylated hemoglobin, type A1C (HbA1c), estimated glomerular filtration rate (eGFR), serum creatinine, human epididymis protein 4 (HE4), and troponin I peak levels. In addition, the Global Registry of Acute Coronary Events (GRACE) score at discharge was calculated (10) for subsequent comparison with our model's prediction ability.

### Model establishment and validation

We used univariate and multivariate logistic regression models to identify the relationship between the variables and new-onset HF. We preliminarily detected potential risk factors using univariate analysis. Potential risk factors (*P* < 0.05) were further analyzed using multicollinearity analysis. If there were variables with a tolerance lower than 0.2 or a variance inflation factor (VIF) higher than 5, we incorporated more meaningful variables based on the empirical screening into the multivariable analysis. Receiver operating characteristic curves were used to detect the relevant cutoff values for selected continuous potential risk predictor variables. To facilitate clinical use, we converted these meaningful continuous variables into binary variables according to the cutoff points. Multivariate logistic regression was performed to build a stepwise nomogram model for new-onset HF within 12 months after ACS. The nomogram was built using forward stepwise method with a threshold of *P* < 0.1 based on the Akaike information criterion. We evaluated the discriminative ability of the model using receiver operating characteristic (ROC) curve analysis and detected the calibration capability between the model probability curve and the ideal calibration curve using a calibration curve. Later, we verified the accuracy of the model by 10-fold cross-validation and evaluated the clinical usefulness of the prognostic nomogram model using decision curve analysis (DCA).

### Statistical analysis

Means (standard deviation) or medians (interquartile range) were used for continuous variables, and numbers and percentages were used for categorical variables. The associations between HF and variables were tested using Student's t-test, Mann–Whitney U test, *χ*^2^ test, Fisher's exact test, and logistic regression model. Statistical analyses were two-tailed with 95% confidence intervals (CIs). Statistical significance was set at *P* < 0.05. Statistical analyses were performed using IBM SPSS Statistics (version 26.0; SPSS Inc., Chicago, IL, USA) and STATA version 15.0 (StataCorp LLC., College Station, TX, USA).

## Results

### Baseline characteristics

We retrospectively analyzed the clinical data collected from Zhejiang Provincial People's Hospital, summarized in Table 1. Three hundred and nineteen female patients were included in this study, and 245 (76.8%) had not developed new-onset HF after ACS, while 74 (23.2%) had. Overall, fifty-five (74.3%) patients of the with-HF group were over 65 years old, and 54 (73.0%) had a history of type 2 diabetes, with 50 (67.6%) patients taking oral antidiabetics or insulin. Patients in the with-HF group had lower LVEF, hemoglobin, HDL-C, and eGFR at admission, but higher levels of HbA1c, creatinine, HE4, and troponin I peak. The number of patients in the with-HF group with coronary artery stenosis ≥50% with 2- or 3-vessel disease was more than that in the without-HF group. Forty (54.1%) patients who had developed new-onset HF experienced myocardial infarction at that time, while 55 (74.3%) had PCI performed. Patients in the with-HF group had a higher proportion rate of beta blockers at discharge. Besides, the mean GRACE score of patients with-HF group was significantly higher.

**Table 1:** Patient characteristics.

Variables	Total	Without HF	With HF	***P***-value
	(*n* = 319)	(*n* = 245)	(*n* = 74)	
**Basic characteristics**				
Age, yrs, *n* (%)				0.001
≤ 65	135 (42.3)	116 (47.3)	19 (25.7)	
> 65	184 (57.7)	129 (52.7)	55 (74.3)	
BMI, kg/m^2^, mean (SD)	23.7 (21.5–26.0)	24.0 (21.4–26.5)	23.0 (21.5–25.0)	0.111
Current smoking, *n* (%)	2 (0.6)	2 (0.8)	0 (0.0)	1.000
Alcohol consumption, *n* (%)	9 (2.8)	2 (2.7)	7 (2.9)	1.000
Initial pulse, bpm, Me (IQR)	76.0 (69.0–84.0)	76.0 (68.0–83.5)	77.0 (72.0–86.0)	0.262
Initial SBP, mmHg, Me (IQR)	138.0 (128.0–154.0)	138.0 (128.0–153.0)	138.5 (127.0–154.5)	0.786
Initial DBP, mmHg, mean (SD)	76.0 (70.0–84.0)	77.0 (70.0–84.0)	74.0 (69.8–81.0)	0.282
**Medical history**				
Hypertension, *n* (%)	221 (69.3)	167 (68.2)	54 (73.0)	0.432
Type 2 diabetes, *n* (%)	159 (49.8)	105 (42.9)	54 (73.0)	< 0.001
**Medications on admission**				
ACEI/ARB, *n* (%)	72 (22.6)	52 (21.2)	20 (27.0)	0.295
CCB, *n* (%)	158 (49.5)	120 (49.0)	38 (51.4)	0.721
Oral antidiabetics/Insulin, *n* (%)	147 (46.1)	97 (39.6)	50 (67.6)	< 0.001
LVEF, %, Me (IQR)	66.0 (61.0–70.0)	67.0 (62.0–70.0)	60.5 (52.0–68.0)	<0.001
Hemoglobin ≤ 115 g/L, *n* (%)	62 (19.4)	39 (15.9)	23 (31.1)	0.004
Total Cholesterol, mmol/L, Me (IQR)	4.4 (3.5–5.2)	4.4 (3.5–5.2)	4.3 (3.4–5.1)	0.322
Triglycerides, mmol/L, Me (IQR)	1.4 (1.0–1.8)	1.4 (0.9–1.8)	1.4 (1.1–1.7)	0.261
HDL-C, mmol/L, Me (IQR)	1.1 (1.0–1.3)	1.2 (1.0–1.3)	1.0 (0.9–1.3)	0.001
LDL-C, mmol/L, Me (IQR)	2.4 (1.8–3.2)	2.4 (1.8–3.2)	2.2 (1.5–3.0)	0.321
HbA1c > 6.0%, *n* (%)	123 (38.6)	83 (33.9)	40 (54.1)	0.002
eGFR, mL/(min·1.73 m^2^), *n* (%)				< 0.001
> 90.0	162 (50.8)	147 (60.0)	15 (20.3)	
60.1–90.0	141 (44.2)	95 (38.8)	46 (62.2)	
45.0–60.0	16 (5.0)	3 (1.2)	13 (17.6)	
Creatinine, µmol/L, Me (IQR)	67.9 (62.0–74.7)	65.1 (61.0–71.7)	77.6 (70.0–88.4)	< 0.001
HE4, pmol/L, Me (IQR)	62.2 (51.6–81.2)	58.9 (49.9–67.9)	92.7 (70.9–120.8)	< 0.001
Troponin I peak > 0.050 µg/L, *n* (%)	126 (39.5)	81 (33.1)	45 (60.8)	< 0.001
**Coronary anatomy, *n* (%)**				< 0.001
No lesions/Stenosis	73 (22.9)	70 (28.6)	3 (4.1)	
One-vessel coronary artery disease	144 (45.1)	116 (47.3)	28 (37.8)	
Two-vessel coronary artery disease	59 (18.5)	41 (16.7)	18 (24.3)	
Three-vessel coronary artery disease	19 (6.0)	8 (3.3)	11 (14.9)	
Left main	24 (7.5)	10 (4.1)	14 (18.9)	
**MI**	58 (18.2)	18 (7.3)	40 (54.1)	< 0.001
**PCI**	198 (62.1)	143 (58.4)	55 (74.3)	0.013
**Medications at discharge**				
Aspirin	284 (89.0)	218 (89.0)	66 (89.2)	0.960
Clopidogrel	244 (76.5)	184 (75.1)	60 (81.1)	0.288
Ticagrelor	22 (6.9)	16 (6.5)	6 (8.1)	0.639
ACEI/ARB	159 (49.8)	121 (49.4)	38 (51.4)	0.767
Beta-blocker	218 (68.3)	160 (65.3)	58 (78.4)	0.034
Statins	315 (98.7)	243 (99.2)	72 (97.3)	0.201
Thiazide diuretic	24 (7.5)	21 (8.6)	3 (4.1)	0.197
SGLT2i	7 (2.2)	5 (2.0)	2 (2.7)	0.665
**GRACE score**	105.0 (88.0–121.0)	103.0 (87.0–117.0)	121.5 (96.0–131.0)	< 0.001

Note: Me, median; IQR, interquartile range; BMI, body mass index; ACEI, angiotensin-converting enzyme inhibitor; ARB, angiotensin receptor blocker; CCB, calcium channel blocker; bpm, beats per minute; SBP, systolic blood pressure; DBP, diastolic blood pressure; SD, standard deviation; LDL-C, low-density lipoprotein cholesterol; HDL-C, high-density lipoprotein cholesterol; HbA1c, glycosylated hemoglobin, type A1C; eGFR, estimated glomerular filtration rate; HE4, human epididymal protein 4; LVEF, left ventricular ejection fraction; MI, myocardial infarction; PCI, percutaneous coronary intervention; SGLT2i, sodium-glucose cotransporter-2 inhibitor; GRACE score, Global Registry of Acute Coronary Events score.

### Potential risk factors for developing new-onset HF after ACS among women

Table 2 presents the results of the univariate logistic regression analyses performed to test the relationship between variables and new-onset HF. Multicollinearity analyses were performed to assess multicollinearity between variables. The risk of HF among female patients was associated with higher prevalence of type 2 diabetes (OR = 3.600, 95%CI, 2.032–6.379, *P* < 0.001), use of oral antidiabetics or insulin (OR = 3.179, 95%CI, 1.823–5.509, *P* < 0.001), morbidity of MI (OR =, 14.837 95%CI, 7.647–28.787, *P* < 0.001), and proportion of performed PCI (OR = 2.065, 95%CI, 1.156–3.688, *P* = 0.014). In addition, the risk of HF among patients with HbA1c > 6.0% was higher than that of those with HbA1c ≤ 6.0% (OR = 2.296, 95%CI, 1.354–3.894, *P* = 0.002). Moreover, the continuous variables of LVEF, HDL-C, creatinine, and HE4 levels were potential predictors of 12-month incidence of HF in the univariate analysis (*P* < 0.001); the categorical variables of age, eGFR, troponin I peak, hemoglobin, and the number of coronary arteries showed a statistically significant difference between the without-HF and with-HF groups (*P* < 0.05). Multicollinearity analyses revealed that the tolerances of the history of diabetes and antidiabetic drugs or insulin prescriptions were smaller than 0.2, and the VIFs were larger than 5, confirming that the regression models were affected by the presence of multicollinearity. Considering that diabetes mellitus may be regarded as equivalent to coronary heart disease (12) and one of the most important risk factors for cardiovascular disease (13), we incorporated type 2 diabetes into the multivariate analysis.

**Table 2:** Potential independent predictors for new-onset HF in female patients after acute coronary syndrome.

Predictors	Univariate analysis			Multicollinearity analysis	
	OR	95% CI	***P***-value	Tolerance	VIF
Age, yrs			0.001	0.836	1.196
≤ 65	Ref	-			
> 65	2.603	1.459–4.643			
Type 2 diabetes	3.600	2.032–6.379	< 0.001	0.121	8.249
Oral antidiabetics/Insulin	3.179	1.823–5.509	< 0.001	0.133	7.504
LVEF, %	0.891	0.855–0.928	< 0.001	0.822	1.217
Hemoglobin ≤ 115 g/L	2.382	1.308–4.339	0.005	0.858	1.166
HDL, mmol/L	0.131	0.041–0.416	0.001	0.897	1.115
HbA1c > 6.0%	2.296	1.354–3.894	0.002	0.706	1.417
eGFR, mL/(min·1.73 m^2^)			< 0.001	0.267	3.751
> 90.0	Ref	-			
60.1–90.0	4.745	2.509–8.975			
45.0–60.0	42.467	10.865–165.982			
Creatinine, µmol/L	1.096	1.066–1.126	< 0.001	0.246	4.060
HE4, pmol/dL	1.056	1.041–1.071	< 0.001	0.476	2.099
Troponin I peak > 0.050 µg/L	3.142	1.836–5.377	< 0.001	0.581	1.721
**Coronary anatomy, *n*(%)**			< 0.001	0.672	1.488
No lesions/Stenosis	Ref	-			
One-vessel coronary artery disease	5.632	1.651–19.212			
Two-vessel coronary artery disease	10.244	2.844–36.902			
Three-vessel coronary artery disease	32.083	7.367–139.722			
Left main	32.667	7.959–134.076			
**MI**	14.837	7.647–28.787	< 0.001	0.609	1.641
**PCI**	2.065	1.156–3.688	0.014	0.707	1.415
Beta-blocker at discharge	1.926	1.043–3.554	0.036	0.931	1.074

OR, odds ratio; CI, confidence interval; HDL-C, high-density lipoprotein cholesterol; HbA1c, glycosylated hemoglobin, type A1C; eGFR, estimated glomerular filtration rate; HE4, human epididymal protein 4; LVEF, left ventricular ejection fraction; MI, myocardial infarction; PCI, percutaneous coronary intervention; VIF, variance inflation factor.

### A prognostic nomogram for 12-month incidence of HF

**Figure 1:**
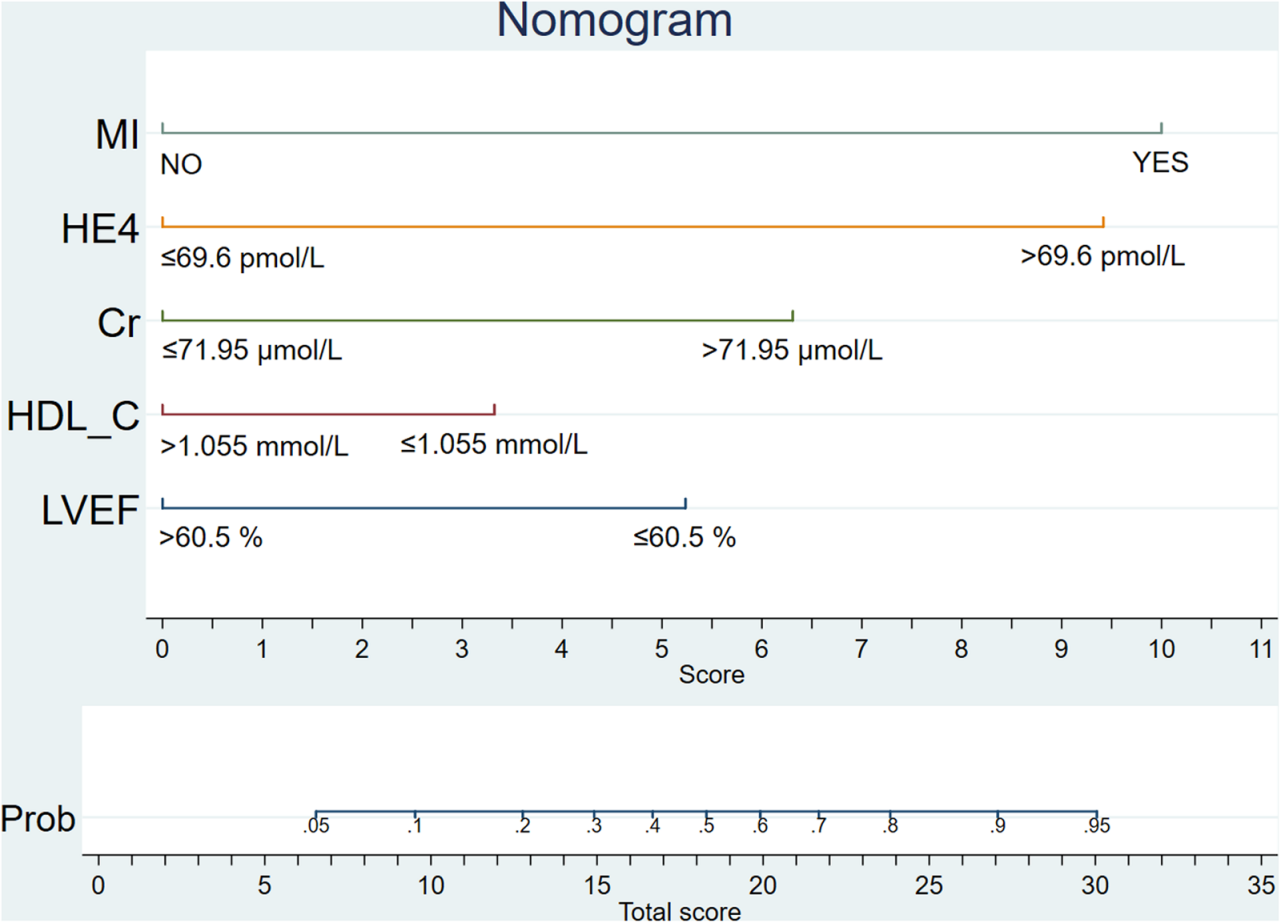
Prognostic nomogram.

**Figure 2:**
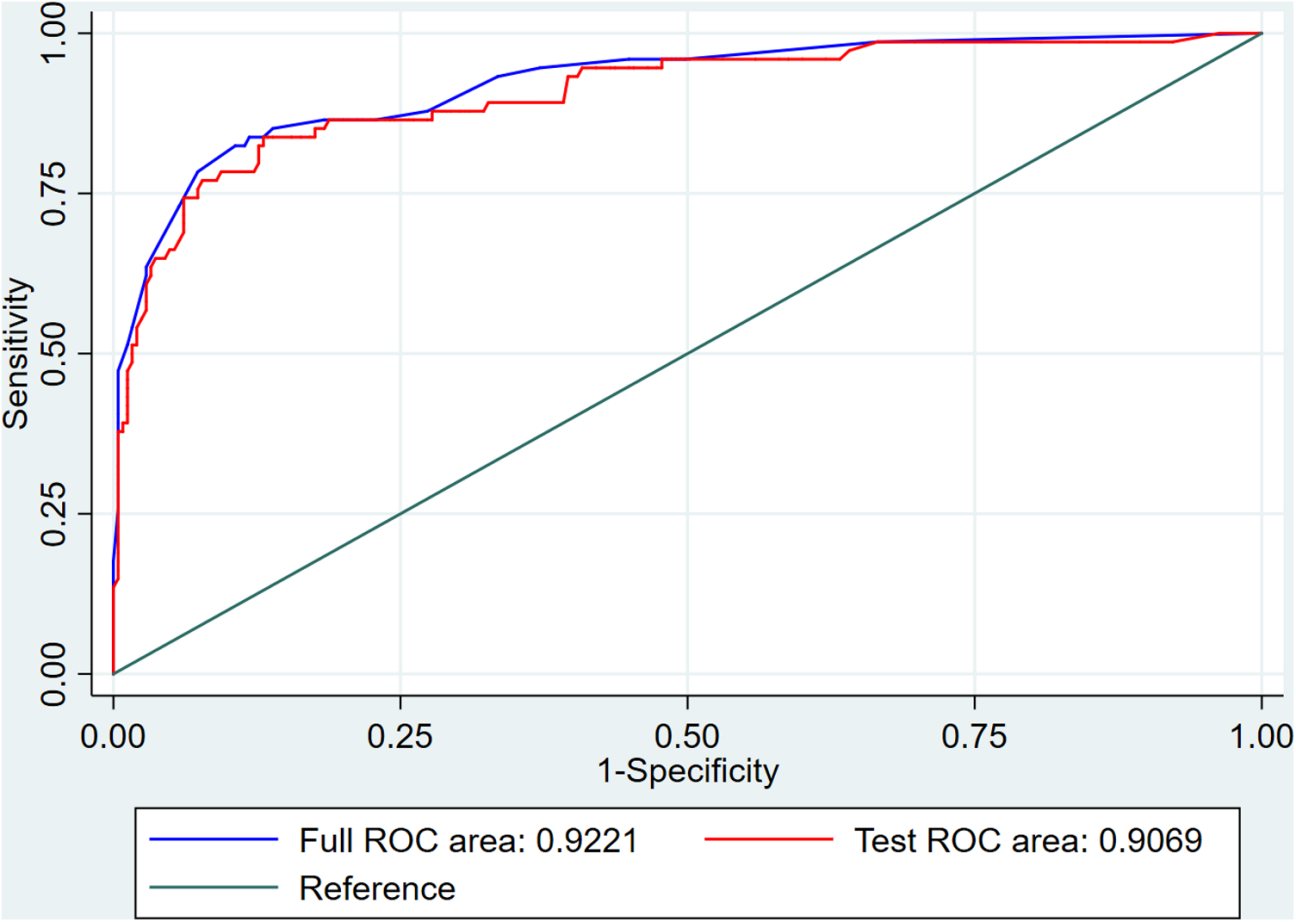
Receiver operating characteristic curve.

To facilitate the convenience of the model, we dichotomized the standard cutoff points for continuous variables, including LVEF (cutoff value = 60.5%), HDL-C (cutoff value = 1.055 mmol/L), creatinine (cutoff value = 71.95 µmol/L), and HE4 (cutoff value = 69.6 pmol/L). Fifteen clinical factors were analyzed to test their relationships with HF using a multivariate logistic model following forward stepwise process and five variables were filtered out from the initial 15: LVEF, HE4, creatinine, HDL-C, and MI (Table 3). The incidence rate of HF could be evaluated using the stepwise nomogram shown in Figure 1. The performance of the model was estimated using ROC curve analysis, and the C-index was 0.922 (sensitivity, 83.8%; specificity, 88.2%), indicating a great diagnostic performance (Figure 2). The calibration *χ*^2^ statistic for the models was 7.32, indicating an excellent goodness-of-fit (*P* = 0.292). And its performance of prediction ability was better than that of GRACE score (AUC = 0.708, sensitivity = 63.5%, and specificity = 74.7%) (Supplementary Figure S1).

**Table 3:** Multivariate model for predicting new-onset heart failure in female patients after acute coronary syndrome.

	Coef.	OR	95% CI	***P***-value
LVEF ≤ 60.5%	1.311	3.712	1.622–8.496	0.002
HE4 > 69.6 pmol/L	2.360	10.588	4.633–24.200	<0.001
Cr > 71.95 µmol/L	1.581	4.858	2.198–10.739	<0.001
HDL-C ≤ 1.055 mmol/L	0.832	2.299	1.053–5.020	0.037
MI	2.506	12.252	4.918–30.519	<0.001

Coef, coefficient; OR, odds ratio; CI, confidence interval; LVEF, left ventricular ejection fraction; HE4, human epididymal protein 4; Cr, creatinine; HDL-C, high-density lipoprotein cholesterol; MI, myocardial infarction.

### Nomogram model verification

**Figure 3:**
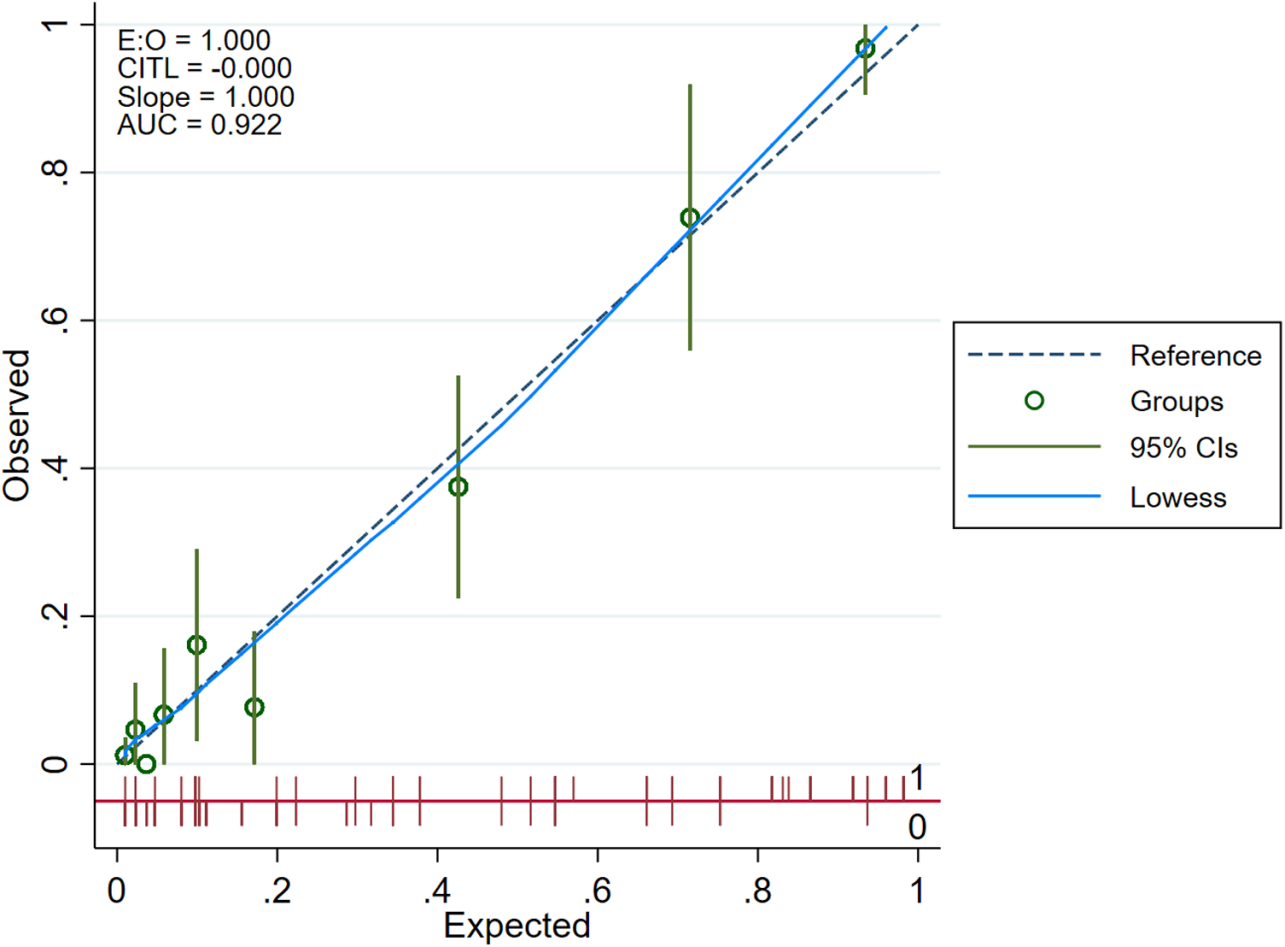
Calibration curve.

**Figure 4:**
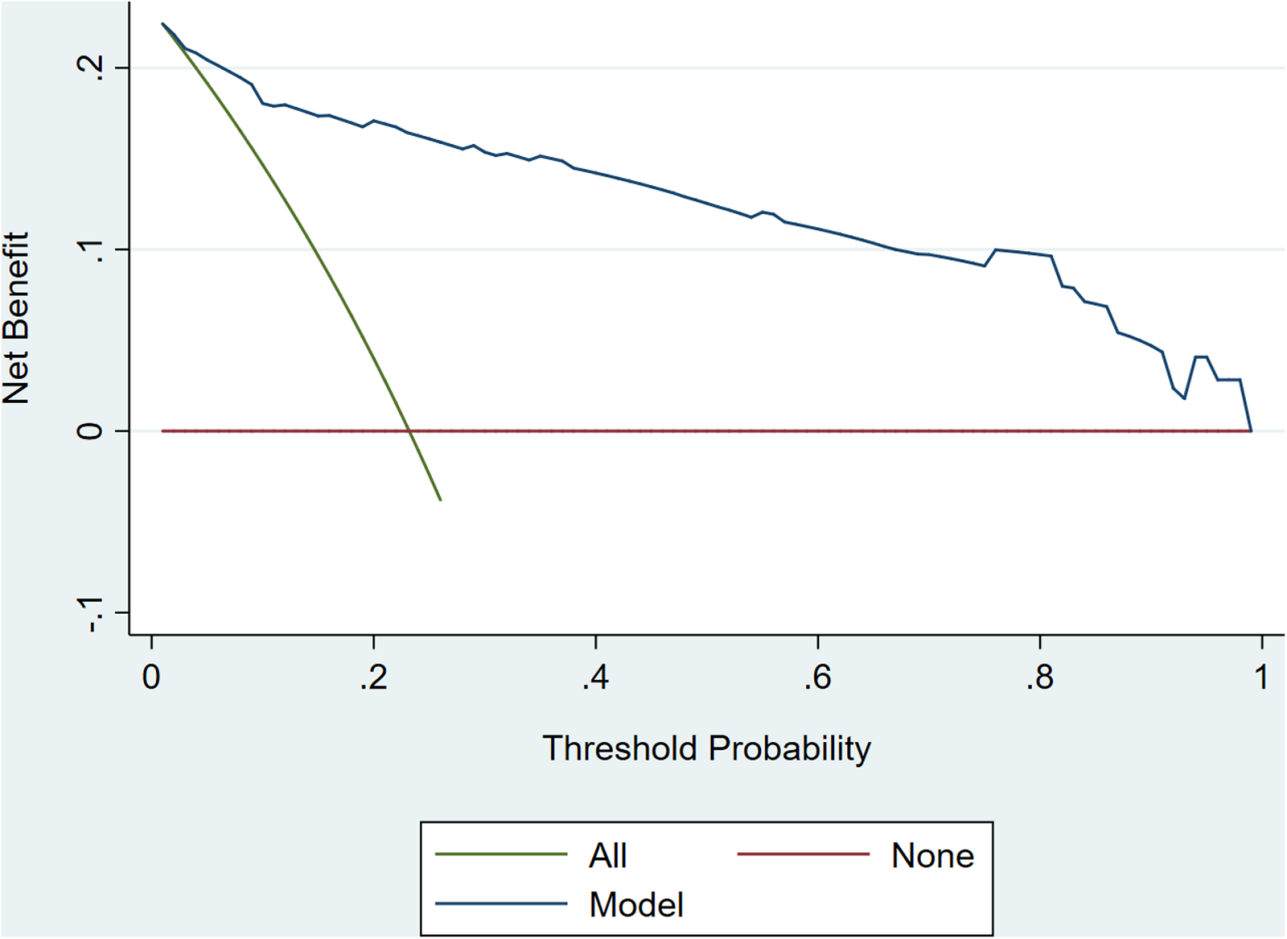
Decision curve analyses.

The internal 10-fold cross-validation verification showed that the stepwise nomogram could accurately predict the C-index of the incidence of HF among female patients within 12 months after ACS, which was 0.907. Furthermore, the calibration curve demonstrated high consistency between the predicted survival probability and actual survival proportion (Figure 3), which was assessed by the Hosmer–Lemeshow statistic (*P* = 0.778). Additionally, decision curve analysis showed great positive net benefits in the model under a threshold probability in the primary cohort (Figure 4), indicating the potential beneficial clinical impact of the model.

## Discussion

Our study analyzed 15 potential independent risk predictors of new-onset HF in 319 female patients with ACS who underwent CAG in Zhejiang Provincial People's Hospital. Seventy-four female patients developed new-onset HF in our study, accounting for 23.2% of all participants with ACS. The following 5 independent risk predictors were screened using stepwise regression for the nomogram: incident MI, LVEF ≤ 60.5%, HE4 > 69.6 pmol/L, HDL-C ≤ 1.055 mmol/L, and creatinine > 71.95 µmol/L. Furthermore, an easy-to-use prediction nomogram was developed for the first time in this study. And our findings of the study are hypothesis generating for future studies. Encouragingly, the model presented an excellent performance in predicting the incidence of new-onset HF (AUC = 0.922, sensitivity = 83.8%, and specificity = 88.2%) and was validated internally using 10-fold cross-validation with AUC = 0.907. Moreover, the developed model presented a superior performance in clinical settings, as shown in the results of the calibration curve and decision curve analysis.

Compared with other models, our model showed some specific strengths. Three risk stratification tools are available. The GRACE score (10) was built to assess the risk of patients who experience ACS with or without STEMI, including future all-cause mortality and MI. Several GRACE risk scores have been established for different patient groups and for predicting different outcomes (14–17). The Thrombolysis in Myocardial Infarction (TIMI) score (18, 19), based on seven clinical variables, is one of the most widely used risk evaluation tools. Nevertheless, the TIMI risk score for UA and NSTEMI validated in a trial (18) showed that the usefulness for prognosis was poor in a real-world dataset of 1-year (20). Subsequently, history, ECG, age, risk factors, and troponin (HEART score) (21), a predictor of outcome in patients with chest pain, outperformed both GRACE and TIMI scores in identifying low-risk ACS (22–24).

Although these risk scores were validated, they did not include important factors. For example, as we emphasized, sex differences cannot be ignored in the prognosis of ACS, especially for HF onset; however, women are often under-represented in large clinical trials (25). Some new factors might contribute to the identification of high-risk patients that were not included in these risk scores. In addition, these risk scores mainly enrolled people from Western countries, and their prognostic study in Asian populations was not sufficiently detailed. Moreover, focusing on the occurrence probability of major adverse cardiovascular and cerebrovascular diseases would weaken the ability to predict the risk of HF onset, an important and high-incidence complication after ACS. Some risk scores were apparently inconvenient to use, with too many factors required. Finally, these tools are mainly used for risk assessment of short-term prognosis.

Considering these shortcomings, our model has attempted to address these issues. First, specifically targeting female patients with ACS is one of the most significant strengths of our nomogram model, which can more accurately assess the incidence of HF in women. The main endpoint of the present study was new-onset HF, which might further refine the management and prognosis of female patients after ACS. Furthermore, all analyses were conducted after adjusting for confounding factors, which were selected from the variables considered reasonable confounders. In our study, we considered all the general cardiovascular risk factors mentioned in the Framingham prediction model (26), including age, SBP, antihypertensive drug use (ACEI or ARB and CCB in our study), TC and HDL-C, smoking status, and diabetes mellitus. Other important factors were also considered. For example, a study conducted by Núñez et al. (2) reported that lower baseline LVEF was associated with a higher risk of new-onset HF, whereas women displayed a higher risk in certain ranges of preserved LVEF, which partly explains our experiment. We identified HE4, a protein that reflects ongoing cardiac fibrosis (27), as a meaningful factor in predicting HF onset, which was consistent with other studies (28, 29). Kumar et al. (30) conducted a study showing that the risk of new-onset HF after ACS was closely related to MI, which remains the most common cause of HF (31). Consistent with previous studies, which reported that plasma creatinine was associated with an increased risk of HF (32, 33), creatinine played an important role in our prediction model. A higher creatinine level reflected a greater prevalence of atherosclerosis and risk factors of aging, as described in another study (34). Therefore, knowledge about HF would make an important contribution to developing new guidelines to reduce its incidence among women.

However, our study was subject to inherent limitations associated with retrospective analysis. First, selection or recall bias was possible in our study. Second, although our model adjusted for confounders and mediators, several confounders that were not included might play an important role, such as other undetected cardiac biomarkers. Third, to clarify the good discrimination and calibration of the model through internal verification, the model requires external validation before we can determine its applicability to other patient populations. Finally, the results of our study might be weakened because of the small sample size. Therefore, we will address these limitations in subsequent prospective studies.

In conclusion, HF is a common complication in patients with ACS, particularly in women. LVEF, HE4, HDL-C, creatinine, and MI were independent risk factors for new-onset HF after ACS among female patients. The novel nomogram that we developed can identify genuine high-risk patients and facilitate communication between female patients and physicians. The prediction model may provide an important early warning for high risk of HF in female patients with ACS.

## Data Availability

The original contributions presented in the study are included in the article/**Supplementary Material**, further inquiries can be directed to the corresponding author/s.
