# Supplementary figures and images for: Nomogram to predict the incidence of new-onset heart failure after acute coronary syndrome among women
Source: Front Cardiovasc Med. 2023 Mar 24;10:1131813. doi: 10.3389/fcvm.2023.1131813 (PMC10080589; doi:10.3389/fcvm.2023.1131813)

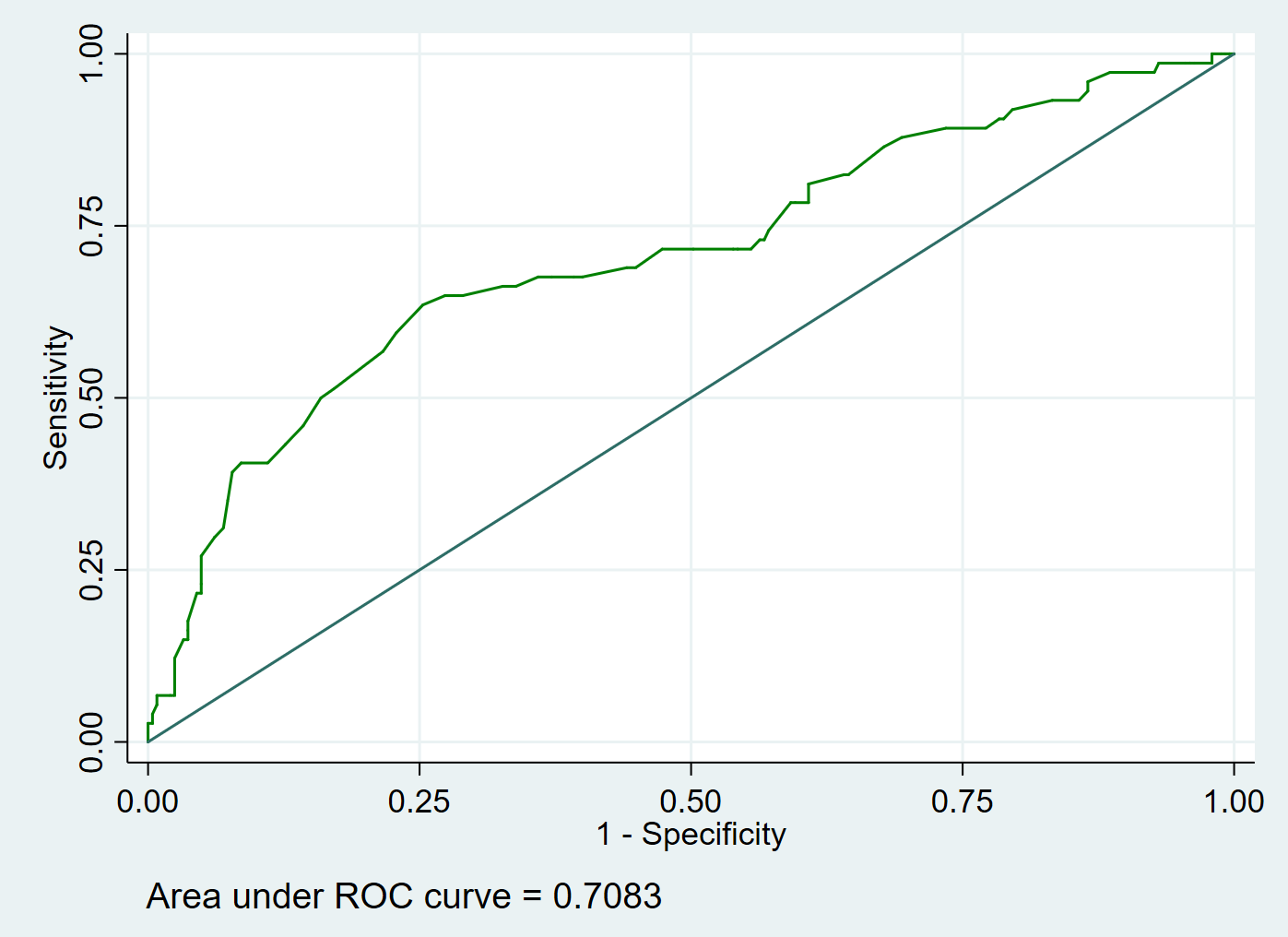

Supplement: Supplementary file 2 [file Image1.tif]
